# A Structural Analysis of Eukaryotic Membrane Evolution

**DOI:** 10.1371/journal.pbio.0020428

**Published:** 2004-11-02

It took nearly 200 years for biologists to redefine the plant/animal dichotomy set up by Linnaeus in 1758. Among the defining traits used in the new five kingdom model of the 20th century was the presence of a nucleus. Possession of a nucleus is one of the chief characteristics that earns an organism, even a single-celled organism, the name of eukaryote. Those not similarly blessed are prokaryotes. Biologists today classify life into three domains (of which microbes lay claim to two), yet the evolution of many fundamental features of eukaryotic biology remains a mystery.

A pivotal moment in the evolution of early eukaryotes was the emergence of elaborate, interconnected membrane-bound compartments that make up the Golgi apparatus, endoplasmic reticulum, and nuclear envelope. The nuclear envelope, with its inner and outer membrane, forms a barrier between the cytoplasm and nucleus. Embedded in this envelope are nuclear pore complexes (NPCs), massive (over 400 subunits), cylindrically shaped protein assemblies that connect the outer and inner nuclear membranes via sharply curved sections of pore membranes. The NPC's central ring-like structure is sandwiched between a cytoplasmic ring, with fibrils extending into the cytoplasm, and a nuclear ring, with a “basket” extending into the nucleoplasm. NPCs police traffic flow between the nucleus and cytoplasm, routinely allowing entry to small molecules while providing only selective passage to macromolecules.[Fig pbio-0020428-g001]

**Figure pbio-0020428-g001:**
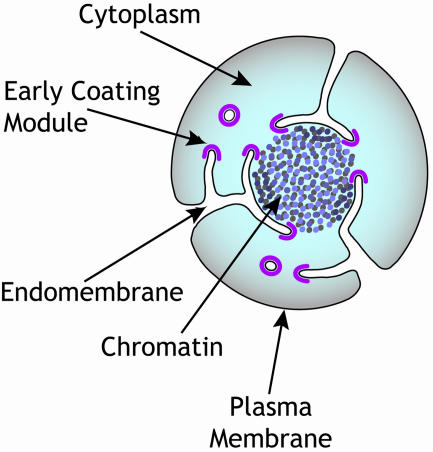
Early eukaryote?

How eukaryotes evolved complex membrane-mediated trafficking systems from their stripped down prokaryotic contemporaries is a fundamental question in biology. Michael Rout's team investigates one aspect of eukaryotic evolution—the origin and evolution of NPC proteins (nups)—by examining the structure of nups. In a new study, Rout and colleagues report the structure of a core building block of the NPC in yeast, and propose how the complex could have evolved from organisms with no such system.

The researchers first tackled the structures of the seven protein components of a core NPC subcomplex, called the yNup84 subcomplex in yeast (and the vNup107-160 subcomplex in vertebrates). Rout and colleagues used algorithms that predict secondary structures to generate three-dimensional models of the component nups. Each nup, they found, consists mostly of either repeating alpha helixes (in an alpha-solenoid fold), zigzagging beta sheets (in a beta-propeller fold), or a distinctive arrangement of an amino-terminal beta-propeller followed by a long stretch of alpha-solenoid. Next, the authors compared the structural conformations of the homologous nups found in humans and plants, and showed that the overall architecture of the subcomplex has been conserved throughout eukaryotic evolution.

A search for evidence of the distinctive propeller/solenoid arrangement in other organisms shed light on the function and origin of the yNup87/vNup107-160 subcomplex. Neither bacterial nor archaebacterial proteins contain such an arrangement; it appears to exist only in eukaryotes. Moreover, proteins containing this arrangement function only as components of the coated vesicle complexes that operate in intracellular vesicular transport systems or as part of the NPC. That these complexes are linked by common architecture, the authors argue, suggests an “intimate connection between vesicle coating complexes and the yNup87/vNup107-160 subcomplex.” It's likely that both complexes function in curving membranes: when components of this subcomplex are disrupted in yeast, NPCs form abnormal clusters that impair nuclear membrane interactions.

How did this shared molecular architecture evolve? Rout and colleagues propose that both nups and vesicle coating complexes developed from a common early eukaryotic ancestor—a primitive coating component with a simplified version of the repetitive folds described here. This molecular carpenter specialized in carving and remodeling membranes, and was repurposed to support the many specialized functions that facilitate molecular transport through the elaborately connected, highly specialized internal membrane systems of the modern eukaryote.

